# Supplementary material for: LC-MS/MS Profiling of Post-Transcriptional Modifications in Ginseng tRNA Purified by a Polysaccharase-Aided Extraction Method
Source: Biomolecules. 2020 Apr 17;10(4):621. doi: 10.3390/biom10040621 (PMC7226401; doi:10.3390/biom10040621)
Supplement: Supplementary file 1 [file biomolecules-10-00621-s001.pdf]

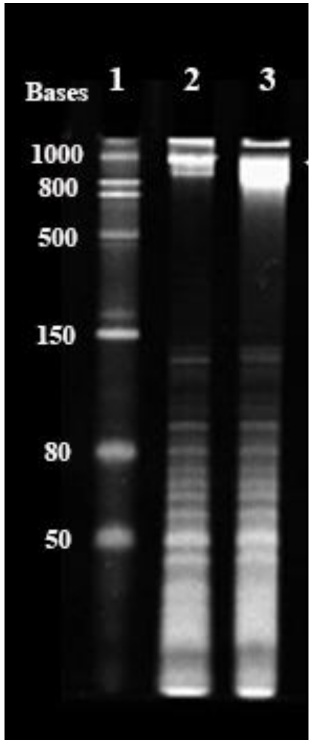

Figure 1. Urea-PAGE analysis of RNA purified from ginseng roots by using TRIzol method (lane 2) and CTAB method (lane 3). Low Range ssRNA Ladder (NEB, USA) ranged from 50 to 1000 mer was used as a molecular weight maker in lane 1.

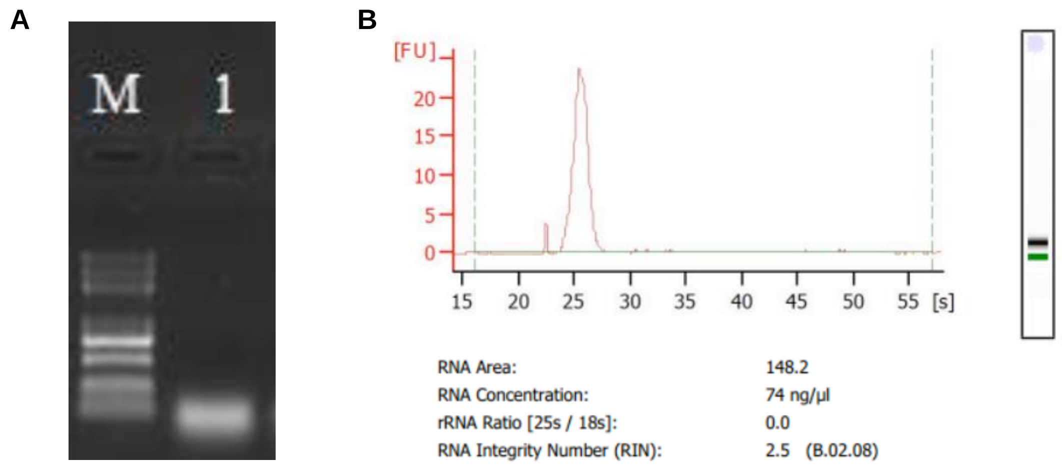

Figure S2: Qualification of cDNA library of tRNA enriched fraction (TEF) by using agarose gel electrophoresis (A) and 2100 bioanalyzer (B).

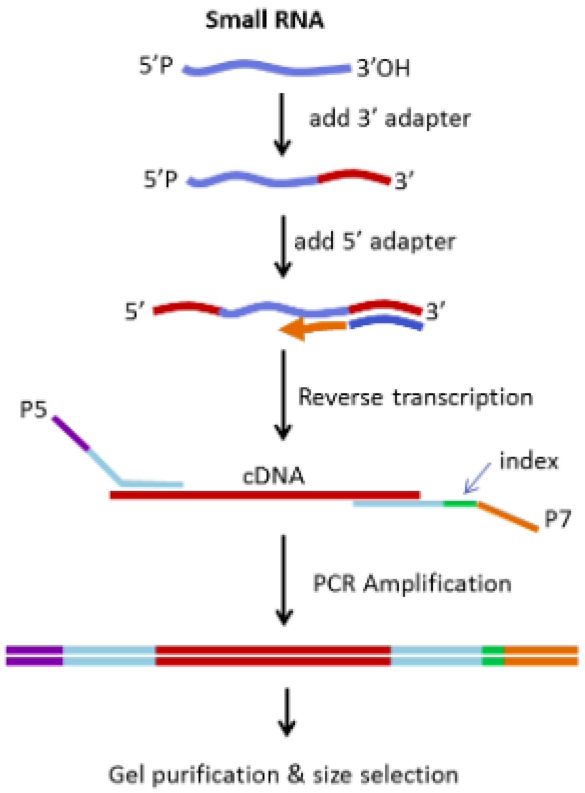

Figure S3: tRNA enriched fraction library preparation.

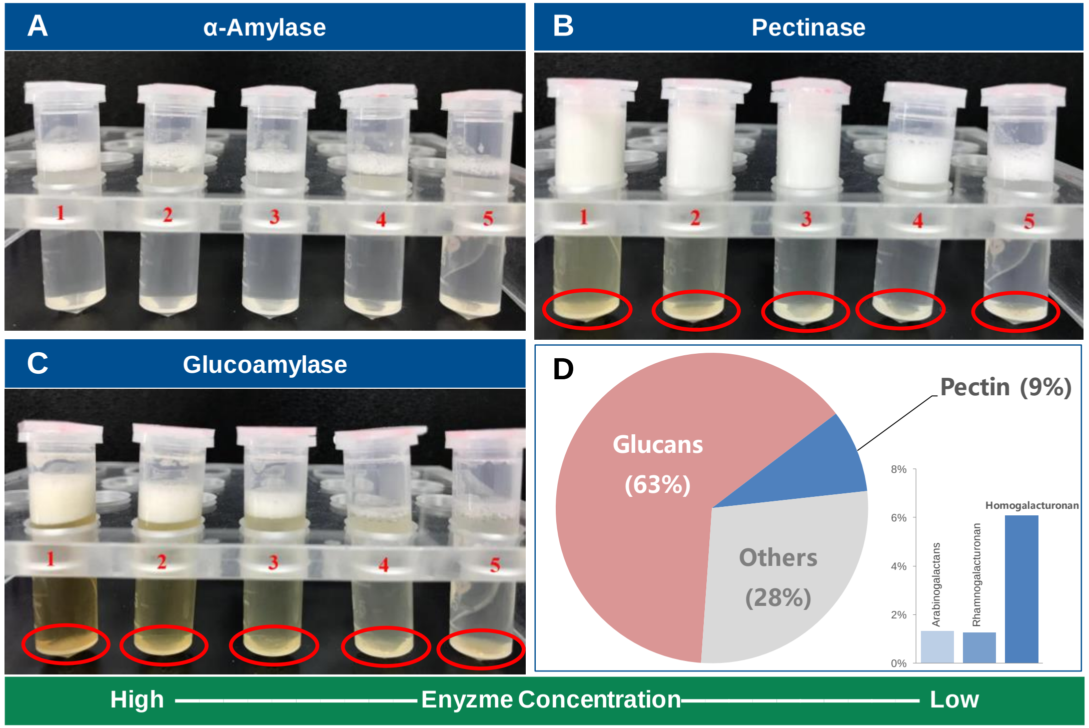

Figure S4: Photos of total RNA isolated from ginseng roots using PARI method with polysaccharases digestion. Polysaccharides of ginseng were digested with corresponding polysaccharases including α-amylase (A), pectinase (B), glucoamylase (C). The amount of each polysaccharases includes 200 mg (tube NO. 1); 100 mg (tube NO. 2); 50 mg (tube NO. 3); 25 mg (tube NO. 4); 12.5 mg (tube NO. 5), respectively. Polysaccharides undissolved were highlighted with red circles. (D) The composition of polysaccharides derived from ginseng roots.

Table S1. Quality measurements of RNA isolated by PARI method using different kinds of polysaccharases.

Polysaccharase	amount (mg)	Sample ID	Raw materials (g)	Yield (μg/g)	RNA Conc. (ng/μl)	A260	A280	260/280	260/230	RIN
α-amylase	200	1	0.2594	96.92	251.4	6.284	3.022	2.1	1.7	7.4
	100	2	0.2082	146.59	305.2	7.63	3.673	2.1	1.8	7.6
	50	3	0.2132	143.20	305.3	7.633	3.639	2.1	2.1	8
	25	4	0.2737	111.98	306.5	7.662	3.676	2.1	1.8	8.5
	12.5	5	0.2056	157.73	324.3	8.108	3.899	2.1	2.1	7.7
Pectinase	200	6	0.2694	36.12	97.3	2.431	4.016	0.6	0.2	1.5
	100	7	0.2753	34.04	93.7	2.342	2.791	0.8	0.2	2.6
	50	8	0.258	26.98	69.6	1.741	1.79	1.0	0.3	2.2
	25	9	0.232	52.54	121.9	3.047	1.535	2.0	1.5	2.4
	12.5	10	0.2124	38.56	81.7	2.044	1.267	1.6	0.6	2.5
Glucoamylase	200	11	0.2096	47.57	99.7	2.492	1.812	1.4	0.3	N/A
	100	12	0.2187	25.70	56.2	1.405	0.971	1.5	0.3	N/A
	50	13	0.2003	15.03	30.1	0.752	0.507	1.5	0.3	2.5
	25	14	0.2024	10.62	21.5	0.538	0.344	1.6	0.3	N/A
	12.5	15	0.233	7.90	18.4	0.459	0.284	1.6	0.3	N/A

Table S2. Quality measurements of RNA isolated with different methods.

Methods	Raw materials (g)	Sample ID	Yield (µg/g)	RNA Conc. (ng/µl)	A260	A280	260/280	260/230	RIN
PARI method	0.2132	3	143.20	305.3	7.663	3.639	2.1	2.07	8.0
TRIzol method	0.2156	16	6.42	12.9	0.322	0.182	1.8	1.4	N/A
CTAB method	0.2009	17	115.72	249.5	6.239	3.076	2.0	2.1	3.1

Table S3. Composition of oligonucleotides from RNase T1 digestion of ginseng $^{tRNAGly(GCC)}$

Theoretical				Experimental				
**Products**	**Location**	**Sequence**	**Mass**	**products**	**Location**	**Sequence**	**RT(min)**	**m/z**
1	G1:G1	pGp	443.204	1				n/a
2	C2:G3	CGp	668.408	2				n/a
3	G4:G4	Gp	363.224	3				n/a
4	A5:G10	AUAUAGp	1963.189	4	A5:G10	AUAUAGp	7.9	980.12 (2-)[a], 1961.25 (1-)
5	U11:G13	UCGp	974.577	5	U11:G13	UCGp	4.7	973.11 (1-)
6-1	A14:G17	AAUGp	1327.811	6	A14:G18	AAU[m7G]Gp	7.5	842.12 (2-), 1685.24 (1-)
6-2	G18:G18	Gp	363.224					n/a
7	U19:G33	UAAAAUUUCUCUUUGp	4739.78	7	U19:G33	[D]AAAAUUUC[Ψ]CUUUGp	12.6	1579.52 (3-), 1184.13 (4-)
8	C34:G38	CCAAGp	1632.01	8	C34:G38	CCAAGp	6.9	814.61 (2-), 1630.23 (1-)
9	G39:G39	Gp	363.224	9				n/a
10	A40:G41	AGp	692.433	10				n/a
11	A42:G44	AAGp	1021.642	11	A42:G44	AAGp	5.9	1020.15 (1-)
12	A45:G47	ACGp	997.617	12	A45:G47	ACGp	5.6	996.14 (1-)
13	C48:G49	CGp	668.408	13				n/a
14	G50:G50	Gp	363.224	14				n/a
15	G51:G51	Gp	363.224	15				n/a
16	U52:G55	UUCGp	1280.746	16	U52:G55	[m5U][Ψ]CGp	6.3	646.07 (2-), 1293.16 (1-)
17	A56:G62	AUUCCCGp	2220.323	17	A56:G62	AUUCCCGp	8.1	738.75 (3-), 1108.64 (2-)
18	C63:G69	CUAUCCGp	2220.323	18	C63:G69	CUAUCCGp	8.5	738.75 (3-), 1108.64 (2-)
19	C70:A74	CCCCA	1487.98	19	C70:A74	CCCCA	5.1	742.62 (2-), 1486.25 (1-)

[a]charge state; n/a, monomers and dimmers were excluded due to their ambiguity.

Table S4: P. *ginseng* genes, primers, amplicon characteristics.

Gene Name	Accession Number	Primer sequence (5’→3’)	Tm (℃)	PCR cycling conditions	Amplicon length(bp)	Reference
cycloartenolsynthase	AB009029	TCATCAGATGGCTCATGGTACG;<br>TCTCCTCCTGTGGGAAATCACC	58.13;<br>59.99	96℃ for 5 min;<br>35 cycles of 96℃ for 30 s, 54℃for 30 s, 72℃ for 1 min;<br>72℃ for 10 min	364	Han J Y, Kwon Y S, Yang D C, et al. Plant and cell physiology, 2006, 47(12): 1653-1662.
β-amyrinsynthase	AB009030	TATCCTGGACACCGAAAGAAGG;<br>CTCCACTTATTTCCTGTTGGGG	58.13;<br>58.13	96℃ for 5 min;<br>35 cycles of 96℃ for 30 s, 53℃for 30 s, 72℃ for 1 min;<br>72℃ for 10 min	445	
β-actin 1	KF699319	TGGCATCACTTTCTACAACG;<br>TTTGTGTCATCTTCTCCCTGTT	53.35;<br>54.40	96℃ for 5 min;<br>35 cycles of 96℃ for 30 s, 49℃for 30 s, 72℃ for 1 min;<br>72℃ for 10 min	109	Liu J, Wang Q, Sun M, et al. PLoS One, 2014, 9(11): e112177.

Table S5: sequence of biotinylated capture DNA probes for purification of ginseng $tRNA^{Gly(GCC)}$

Target	Probe sequence (5’→3’)	Length (mer)	Mass (Da)	Tm (°C)
$tRNA^{Gly(GCC)}$	biotin-TCCTTGGCAAAGAGAAATTTTACCATTCGA	30	9595.50	68

Table S6: Liquid chromatography parameters for oligonucleotides analysis.

**Agilent 1290 infinity UPLC system**	
Parameter	
Column	Waters Acquity OST C18 column (1.7 μm, 2.1 × 100 mm)
Column temperature	60 ℃
Mobile phase A	15 mM TEA and 100mM HFIP in water (pH=8.5)
Mobile phase B	15 mM TEA and 100mM HFIP in 50% methanol/water (v/v)
Gradient	0.00-1.50 min, 2% B<br>1.50-8.30 min, 2%-32% B<br>8.30-16.50 min, 32%-38% B<br>16.50-20.00 min, 38%-42% B
Flow rate	0.2 ml/min

Table S7: Mass spectrometer parameters for oligonucleotides analysis.

**Agilent 6545 QTOF-MS system**	
Parameter	
Ion mode	Negative ion mode
Source	Agilent Dual Jet Stream
Drying gas temperature	320°C
Drying gas flow	12 L/min
Sheath gas temperature	350°C
Sheath gas flow	12 L/min
Nebulizer	35 psig
Capillary voltage	3500 V
Nozzle voltage	1700 V
Fragmentor voltage	220 V
m/z range (MS1)	600-2000
m/z range (MS2)	100-1800

Table S8: Liquid chromatography parameters for nucleoside analysis.

**Agilent 1290 infinity UPLC system**	
Parameter	
Column	Agilent Poroshell 120 HPLC column (2.7 μm, 4.6 × 100 mm)
Column temperature	35 ℃
Mobile phase A	0.1% formic acid in water
Mobile phase B	0.1% formic acid in acetonitrile
Gradient	0.00-4.00 min, 1.5%-4% B<br>4.00-12.00 min, 4%-15% B<br>12.00-18.00 min, 15%-25% B<br>18.00-21.00, 1.5% B
Flow rate	0.4 ml/min

Table S9: Mass spectrometer parameters for nucleoside analysis.

**Agilent 6550 QTOF-MS system**	
Parameter	
Ion mode	Positive ion mode
Source	Agilent Dual Jet Stream
Drying gas temperature	250°C
Drying gas flow	15 L/min
Sheath gas temperature	300°C
Sheath gas flow	11 L/min
Nebulizer	22 psig
Capillary voltage	3500 V
Fragmentor voltage	380 V
m/z range (MS1)	108-650

Table S10: tRNA transcripts in ginseng root determined by NGS.

Isoacceptor	Sequence (5’→3’)
His-GUG	GCGGATGTAGCCAAGTGGATCAAGGCAGTGGATTGTGAATCCACCATGCGCGGGTTCAATTCCCGTCGTTCGCCCCA
Asp-GUC	GGGATTGTAGTTCAATTGGTCAGAGCACCGCCCTGTCAAGGCGGAAGCTGCGGGTTCGAGCCCCGTCAGTCCCGCCA
Gly-GCC	GCGGATATAGTCGAATGGTAAAATTTCTCTTTGCCAAGGAGAAGACGCGGGTTCGATTCCCGCTATCCGCCCCA
Met-CAU	CGCGGAGTAGAGCAGTTTGGTAGCTCGCAAGGCTCATAACCTTGAGGTCACGGGTTCAAATCCTGTCTCCGCAACCA
Val-GAC	AGGGATATAACTCAGCGGTAGAGTGTCACCTTGACGTGGTGGAAGTCATCAGTTCGAGCCTGATTATCCCTACCA
Gln-UUG	TGGGGCGTGGCCAAGTGGTAAGGCAACGGGTTTTGGTCCCGCTATTCGGAGGTTCGAATCCTTCCGTCCCAGCCA
Pro-UGG	AGGGATGTAGCGCAGCTTGGTAGCGCTTTTGTTTTGGGTACAAAATGTCACGGGTTCAAATCCTGTCATCCCTACCA
Val-UAC	AGGGCTATAGCTCAGTTAGGTAGAGCACCTCGTTTACACCGAGAAGGTCTACGGTCCGAGTCCGTATAGCCCTA
Leu-UAA	GGGGATATGGCGGAATTGGTAGACGCTACGGACTTAAAATCCGTCGACTTTAAAATCGTGAGGGTTCAAGTCCCTCTATCCCCACCA
Asn-GUU	TCCTCAGTAGCTCAGTGGTAGAGCGGTCGGCTGTTAACTGACTGGTCGTAGGTTCGAATCCTACCTGGGGAGCCA
Leu-CAA	GCCTTGGTGGTGAAATGGTAGACACGCGAGACTCAAAATCTCGTGCTAAAGAGCGTGGAGGTTCGAGTCCTCTTCAAGGCACCA
Glu-UUC	GCCCCCATCGTCTAGTGGTTCAGGACATCTCTCTTTCAAGGAGGCAGCGGGGATTCGACTTCCCCTGGGGGTACCA
Gly-UCC	GCGGGTATAGTTTAGTGGTAAAACCCTAGCCTTCCAAGCTAACGATGCGGGTTCGATTCCCGCTACCCGCTCCA
Ser-GCU	GGAGAGATGGCTGAGTGGACTAAAGCGGCGGATTGCTAATCCGCTGTACGAGTTATTCGTACCGAGGGTTCGAATCCCTCTCTTTCCGCCA
Phe-GAA	GTCGGGATAGCTCAGCTGGTAGAGCAGAGGACTGAAAATCCTCGTGTCACCAGTTCAAATCTGGTTCCTGGCACCA
Leu-UAG	GCCGCTATGGTGAAATCGGTAGACACGCTGCTCTTAGGAAGCAGTGCTAGAGCATCTCGGTTCGAGTCCGAGTGGCGGCACCA
Ser-GGA	AGGAGAGATGGCCGAGTGGTTGAAGGCGTAGCATTGGAACTGCTATGTAGGCTTTTGTTTACCGAGGGTTCGAATCCCTCTCTTTCCG
Ser-UGA	GGAGAGATGGCTGAGTGGTTGATAGCTCCGGTCTTGAAAACCGGCATAGTTTTAACAAAGAACTATCGAGGGTTCGAATCCCTCTCTCTCCTCCA
Arg-ACG	GGGCCTGTAGCTCAGAGGATTAGAGCACGTGGCTACGAACCACGGTGTCGGGGGTTCGAATCCCTCCTCGCCCACCA
Cys-GCA	GGCGATATGGCCGAGTGGTAAGGCGGGGGACTGCAAATCCTTTTTTCCCCAGTTCAAATCCGGGTGTCGCCTCCA
Trp-CCA	GCGCTCTTAGTTCAGTTCGGTAGAACGTGGGTCTCCAAAACCCAATGTCGTAGGTTCAAATCCTACAGAGCGTGCCA
Arg-UCU	GCGTCCATTGTCTAATGGATAGGACAGAGGTCTTCTAAACCTTTGGTATAGGTTCAAATCCTATTGGACGCACCA
Tyr-GUA	GGGTCGATGCCCGAGCGGTTAATGGGGACGGACTGTAAATTCGTTGGCAATATGTCTACGCTGGTTCAAATCCAGCTCGGCCCACCA
His-CAU	GCATCCATGGCTGAATGGTTAAAGCGCCCAACTCATAATTGGCGAATTCGTAGGTTCAATTCCTACTGGATGCACCA
Thr-GGU	GCCCTTTTAACTCAGCGGTAGAGTAACGCCATGGTAAGGCGTAAGTCATCGGTTCAAATCCGATAAGGGGCTCCA
Gly-GCC	GCACCAGTGGTCTAGTGGTAGAATAGTACCCTGCCACGGTACAGACCCGGGTTCGTTTCCCGGCTGGTGCACCA
Glu-CUC	TCTTCGCTAGTATATCGGTTAGTATATTCGCCTCTCACGCGAAAGAGCAGGGTTCAACTCCCTGGCGGAGAACCA
Gln-CUG	GGTTCCATGGTCTAGTGGTCAGGACATTGGACTCTGAATCCAGTAACCCGAGTTCAGGTCTCGGTGGAACCTCCA
Met-CAU	AGCGGGGTAGAGTAATGGTCAACTCATCAGTCTCATTATCTGAAGACTACAGGTTCGAATCCTGTCCCCGCCTCCA
Ala-AGC	GGGGATGTAGCTCAGATGGTAGAGCGCTCGCTTAGCATGCGAGAGGTACGGGGATCGATACCCCGCATCTCCACCA
Asp-GUC	GTCGTTGTAGTATAGTGGTAAGTATTCCCGCCTGTCACGCGGGTGACCCGGGTTCGATCCCCGGCAACGGCGCCA
Phe-GAA	GCGGGGATAGCTCAGTTGGGAGAGTGTCAGACTGAAGATCTAAAGGTCACGTGTTTGATCCACGTTCACCGCACCA
Cys-GCA	GGCTAGGTAACATAATGGAAATGTATTGGACTGCAAATCCTGGAATGACGGTTCGACCCCGTCCTTGGCCTCCA
Pro-UGG	CGAGGTGTAGCGCAGTCTGGTCAGCGCATCTGTTTTGGGTACAGAGGGCCATAGGTTCGAATCCTGTCACCTTGACCA
Glu-UUC	GTCCCTTTCGTCCAGTGGTTAGGACATCGTCTTTTCATGTCGAAGACACGGGTTCGATTCCCGTAAGGGGTACCA
Val-AAC	GGTTTCGTGGTGTAGTTGGTTATCACGTCAGCCTAACACACTGAAGGTCTCCGGTTCGAACCCGGGCGAAGCCACCA
Glu-UCC	TCCGTTGTCGTCCAGCGGTTAGGATATCTGGCTTTCACCCAGGAGACCCGGGTTCGTTTCCCGGCAACGGAACCA
Ser-UGA	GGATGGATGTCTGAGCGGTTGGAAGAGTCGGTCTTGAAAACCGAAGTATTGATAGGAATACCGGGGGTTCGAATCCCTCTCCATCCGCCA
Arg-CCU	GCGCCTGTAGCTCAGTGGATAGAGCGTCTGTTTCCTAAGCAGAAAGTCGTAGGTTCGACCCCTACCTGGCGCGCCA
Val-CAC	GTCTGGGTGGTGTAGTCGGTTATCATGCTAGTCTCACACACTAGAGGTCCCCGGTTCGAACCCGGGCTCAGACACCA
Glu-CUC	TCCGTTGTAGTCTAGTTGGTCAGGATACTCGGCTCTCACCCGAGAGACCCGGGTTCAAGTCCCGGCAACGGAACCA
